# *Vibrio* Zinc-Metalloprotease Causes Photoinactivation of Coral Endosymbionts and Coral Tissue Lesions

**DOI:** 10.1371/journal.pone.0004511

**Published:** 2009-02-19

**Authors:** Meir Sussman, Jos C. Mieog, Jason Doyle, Steven Victor, Bette L. Willis, David G. Bourne

**Affiliations:** 1 ARC Centre of Excellence for Coral Reef Studies, and School of Marine and Tropical Biology, James Cook University, Townsville, Queensland, Australia; 2 Australian Institute of Marine Science (AIMS), Townsville MC, Townsville, Queensland, Australia; 3 University of Groningen, Dept. of Marine Bentic Ecology and Evolution, Biological Center, Haren, The Netherlands; 4 Palau International Coral Reef Center (PICRC), Koror, Republic of Palau; University of North Carolina at Chapel Hill, United States of America

## Abstract

**Background:**

Coral diseases are emerging as a serious threat to coral reefs worldwide. Of nine coral infectious diseases, whose pathogens have been characterized, six are caused by agents from the family Vibrionacae, raising questions as to their origin and role in coral disease aetiology.

**Methodology/Principal Findings:**

Here we report on a *Vibrio* zinc-metalloprotease causing rapid photoinactivation of susceptible *Symbiodinium* endosymbionts followed by lesions in coral tissue. *Symbiodinium* photosystem II inactivation was diagnosed by an imaging pulse amplitude modulation fluorometer in two bioassays, performed by exposing *Symbiodinium* cells and coral juveniles to non-inhibited and EDTA-inhibited supernatants derived from coral white syndrome pathogens.

**Conclusion/Significance:**

These findings demonstrate a common virulence factor from four phylogenetically related coral pathogens, suggesting that zinc-metalloproteases may play an important role in *Vibrio* pathogenicity in scleractinian corals.

## Introduction

Coral diseases have emerged over the last decades as a serious threat to coral reefs worldwide [1]–[2], with elevated seawater temperatures [3]–[5] and other anthropogenic stressors [6]–[7] identified as major contributors to marine ecosystem deterioration. Of nine coral infectious diseases, whose pathogens have been characterized by fulfilling Henle-Koch's postulates [8], six are caused by agents from the family Vibrionacae [9]–[12], adding to the many previously characterized *Vibrio* infections of shrimps [13], clams [14] and fish [15], which date back to 1817 [16]. Other coral disease signs in the Caribbean [17]–[18] have also been associated with the presence of *Vibrio* agents. The study of coral disease signs in Zanzibar [19], bleached corals on the Great Barrier Reef (GBR; [20]), black band disease signs on corals in the Gulf of Aquaba (the Red Sea; [21]) and even growth anomalies on Hawaiian corals [22] have all demonstrated significant correlation between disease signs and an elevated abundance of *Vibrio* strains. These newly emerging coral diseases, either caused or associated with members of the Vibrionacae family have sparked a debate on the origin of *Vibrio* pathogens and their role in the aetiology of coral diseases: Are *Vibrio* pathogens the primary causative agents of all these diseases? Are they opportunistic pathogens? Or are they secondary infections to other unknown causes? [23]–[31]

In a recent study [12] we identified two novel *V. coralliilyticus* strains and four additional *Vibrio* pathogens as causative agents of three Indo-Pacific coral white syndromes (WS's). In that study, a link was demonstrated between WS disease signs on corals and the presence of *Vibrio* strains possessing a zinc-metalloprotease gene [12]. Protein homologues of this gene have been identified as key virulence factors of *Vibrio* pathogens of fish [32], shrimp [33], mollusks [34] and humans [35] acting to digest mucin and other connective tissue components, such as collagen IV [36] and fibronectin [37]. These enzymes have also been shown to perturb paracellular barrier functions [38] and cause tissue necrosis [39] including pathogen detachment from epithelial mucus [40]. Ben-Haim et al. [41] suggested that *V. coralliilyticus*, the bleaching agent of the coral *Pocillopora damicornis*, expresses a *V. cholera*–like zinc-metalloprotease, which causes rapid photosystem II (PS II) inactivation of *Symbiodinium* endosymbionts. However, little is known about either the kinetics or the specificity of this reaction, and under which conditions it is likely to occur. Numerous studies have demonstrated that the zinc-metalloprotease gene is present in *Vibrio* pathogenic strains, but also in non-pathogenic strains [12], [42], suggesting that this gene may not be considered an essential virulence factor [39], [43].

In this study we tested this hypothesis and the role of zinc-metalloprotease in the pathogenicity of coral WS's by developing two novel bioassays. *Symbiodinium* cells from four coral hosts at two locations on the GBR were isolated and grown in cultures (Z1–Z4; Table 1) before being exposed in 96 well microtitre plates to bacterial supernatants derived from four coral pathogens (P1–P4; Table 2) that have been characterized as the causative agents of coral WS's on Pacific reefs, *i.e.*, on the GBR (P1), in the Republic of the Marshall Islands (P2) and in Palau (P3–P4; [12]). In order to test the effects of pathogen supernatants on *Symbiodinium* cells living *in hospite*, a second bioassay was developed by rearing juveniles of *Acropora millepora* and infecting them with specific *Symbiodinium* isolates from clades C and D [44]. To test PS II inactivation by pathogen supernatants, this study used an imaging pulse amplitude modulation (iPAM) fluorometer (Walz, Germany) to measure both dark adapted PS II quantum yields, F_v_/F_m_ = (*F*_m_−*F*_o_)/*F*_m_ [45], and light adapted effective PS II quantum yields, ΔF/F_m′_, which estimate *Symbiodinium* PS II activity in either a relaxed or active state, respectively [46]–[48]. Use of the iPAM system allowed up to 96 replicates per analysis of cultured *Symbiodinium* cells and up to 48 replicates per analysis of coral juveniles. From quantum yield values, PS II inactivation (I) was calculated as a proportion, where 1.0 represented 100% PS II inactivation following exposure to bacterial supernatants and four negative controls, including bacterial supernatants, whose proteolytic activity was inhibited by EDTA (Table S1).

**Table 1:** *Symbiodinium* cultures Z1–Z4.

Culture	Clone names^1^	Isolated from	Location	Date	Genback Acc.^1^
Z1-1	*MAEQMI 12*	*Montipora aequituberculata*	Nelly Bay	Jan. 2006	EU567151
Z1-2	*MAEQMI 2*	*Montipora aequituberculata*	Nelly Bay	Jan. 2006	EU567152
Z2-1	*MAEQDR 38*	*Montipora aequituberculata*	Davies Reef	Oct. 2005	EU567155
Z2-2	*MAEQDR 37*	*Montipora aequituberculata*	Davies Reef	Oct. 2005	EU567156
Z2-2	*MAEQDR 2*	*Montipora aequituberculata*	Davies Reef	Oct. 2005	EU567157
Z3-1	ATMI 21	*Acropora tenius*	Nelly Bay	Jan. 2006	EU567160
Z3-2	ATMI 23	*Acropora tenius*	Nelly Bay	Jan. 2006	EU567167
Z3-3	ATMI 48	*Acropora tenius*	Nelly Bay	Jan. 2006	EU567168
Z4-1	AMMI V6	*Acropora millepora*	Nelly Bay	Jan. 2006	EU567158
Z4-2	AMMI V24	*Acropora millepora*	Nelly Bay	Jan. 2006	EU567159
Z4-3	AMMI 18	*Acropora millepora*	Nelly Bay	Jan. 2006	EU567170
Z4-4	AMMI 12	*Acropora millepora*	Nelly Bay	Jan. 2006	EU567174

1 Sequences (∼360 bp) including the ITS-1 rRNA and its flanking regions were submitted to www.ncbi.nih.nlm.gov/Genbank and are identified by clone names and clone numbers.

**Table 2:** *Vibrio* White Syndrome coral pathogens.

	Isolated from	Location	Date	Genbank Acc.^1^	LMG Acc.^2^
P1	*Montipora aequituberculata*	Nelly Bay, GBR	Sep. 2003	EU372917	LMG23696
P2	*Acropora cytherea*	Marshall Islands	Aug. 2004	EU372935	LMG23691
P3	*Pachyseris speciosa*	Nikko Bay, Palau	Feb. 2005	EU372934	LMG23695
P4	*Pachyseris speciosa*	Nikko Bay, Palau	Feb. 2005	EU372931	LMG23693

1 Near full-length 16S rRNA sequences (>1200 bp) were submitted to www.ncbi.nih.nlm.gov/Genbank.

2 Pathogen isolates were submitted to the public collection of BCCM/LMG at the Ghent University, Belgium under the following identifications: P1 = MMS1, P2 = RMI1, P3 = MSP8, P4 = MSP13.

## Results

### *Symbiodinium* culture Z1 is most susceptible to bacterial PS II inactivation

**Figure 1:**
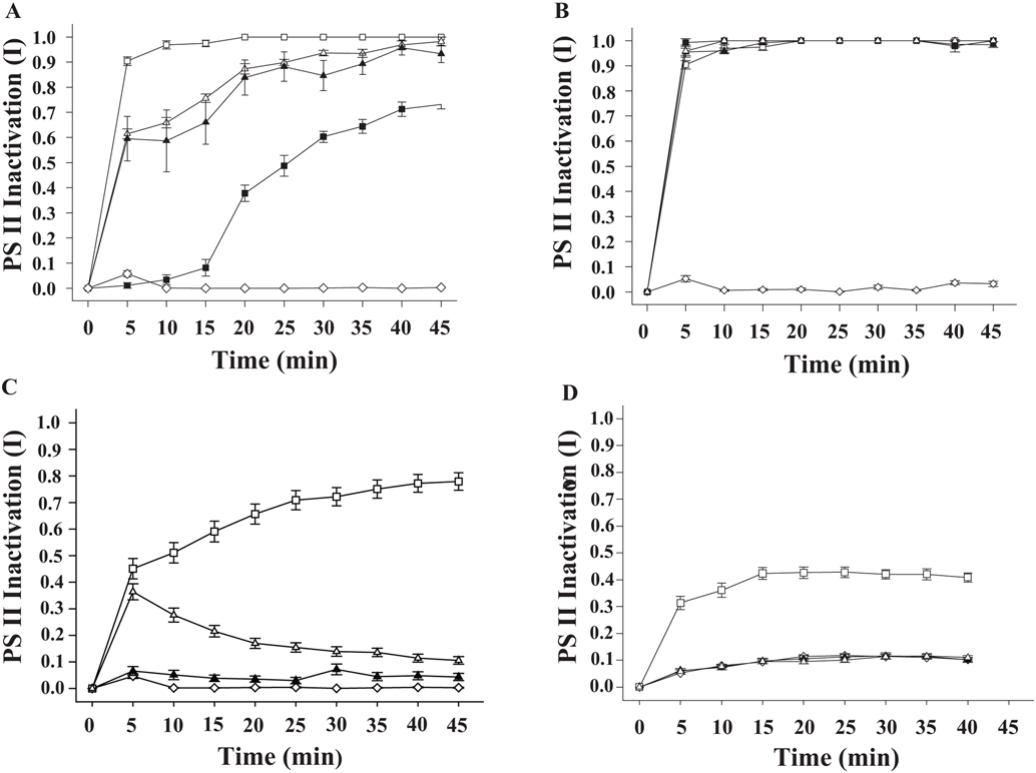
PS II inactivation of *Symbiodinium* cultures by bacterial supernatants. A. PS II inactivation (I; ΔF/F_m′_) by P1 supernatant of *Symbiodinium* cultures Z1 □, Z2 ▴, Z3 ▵, Z4 ▪ and pooled data for Z1–Z4 cultures exposed to dinoflagellate growth medium (F2) ⋄. B. PS II inactivation (I; ΔF/F_m′_) of *Symbiodinium* culture Z1 exposed to pathogen supernatants P1 □, P2 ▪, P3 ▴, P4 ▵ and *Symbiodinium* culture Z1 exposed to dinoflagellate growth medium (F2) ⋄ C. Pooled data for PS II inactivation (I; ΔF/F_m′_) of *Symbiodinium* cultures Z1–Z4 exposed to: pathogen supernatants P1–P4 □, Pathogen supernatants P1–P4 inhibited by incubation with 50 mM EDTA for 1 h at 30°C ▵, a 1∶1 mix of bacterial growth medium (MB) and dinoflagellate growth medium F2 ⋄, Dinoflagellate growth medium (F2) ▴. D. Pooled data for PS II inactivation (I; F_v_/F_m_) of *Symbiodinium* cultures Z1–Z4 exposed to: pathogen supernatants P1–P4 □, Pathogen supernatants P1–P4 inhibited by incubation with 50 mM EDTA for 1 h at 30°C ▵, 1∶1 mix of bacterial growth medium (MB) and dinoflagellate growth medium F2 ⋄, Dinoflagellate growth medium (F2) ▴. 96 microtitre plates were loaded with 2.5×10^5^ *Symbiodinium* cells well^−1^. I; ΔF/F_m′_ was based on measurements of effective light adapted quantum yields. I; F_v_/F_m_ was based on measurements of dark adapted quantum yields. Bars = standard errors. n = 8 measurements per treatment.

**Figure 2:**
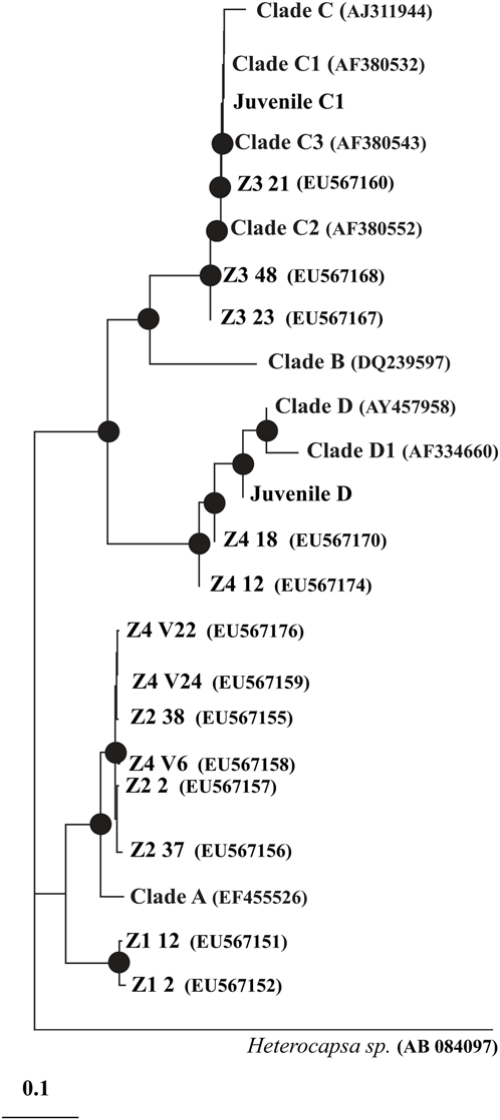
Neighbour joining phylogenetic tree of *Symbiodinium* cultures Z1–Z4. *Symbiodinium* sequences obtained via cloning of PCR products are presented by culture name (*i.e.*, Z1–Z4) followed by clone number and Genbank accession number (in brackets). Clones obtained from *Symbiodinium* cultures used to infect coral juveniles appear as Juvenile C1 and Juvenile D. Reference types representing *Symbiodinium* clades were obtained from authors listed in M&M. Bootstrapping with 1000 replicates was performed and values ≥50% were included for main nodes of the tree.

*Symbiodinium* culture Z1 isolated from the WS susceptible coral host *Montipora aequituberculata* at Nelly Bay, an inshore reef off Magnetic Island in the central GBR, was the most severely affected of the four *Symbiodinium* cultures tested when exposed to P1 supernatant under illumination (p<0.01; Fig. 1A). For *Symbiodinium* culture Z1, inactivation (I) of PS II (measured as light adapted quantum yields) was greater than 95% (mean I (Z1 ΔF/F_m′_) = 0.968±0.016) following exposure to P1 supernatant for 10 min in two independent experiments, and total PS II inactivation resulted after 20 min (Fig. 1A). A significant (∼40%; p<0.0001) difference in mean I was measured between this culture from Nelly Bay and culture Z2 isolated from the same coral species found at Davies Reef, a GBR midshelf reef located less than a 100 km away, where no signs of WS on *M. aequituberculata* have been observed [mean I (Z2 ΔF/F_m′_) = 0.587±0.021 following exposure to P1 supernatant for 10 min in two independent experiments]. The impact of P1 on culture Z3, which was isolated from the coral *Acropora tenuis* at Nelly Bay, where it has not been observed with signs of WS, was similar to its impact on *Symbiodinium* culture Z2 throughout the experiment (p = 0.426). *Symbiodinium* culture Z4 isolated from the coral *Acropora millepora* at Nelly Bay, where it has not been observed with WS signs at this site, was the least affected (∼3%; p<0.01) of all *Symbiodinium* cultures tested in this study [mean I (Z4 ΔF/F_m′_) = 0.034±0.019 following exposure to P1 supernatant for 10 min in two independent experiments]. Control treatments with dinoflagellate growth medium F2 (Fig. 1A) and bacterial growth medium (MB) resulted in limited or no PS II inactivation of the respective cultures [mean I (F2 ΔF/F_m′_) = 0.001±0.001; p<0.01, and mean I (MB ΔF/F_m′_) = 0 following exposure for 10 min in two independent experiments]. Cloning and sequencing the ribosomal RNA (rRNA) internal transcribed spacer 1 (ITS-1) region of *Symbiodinium* from cultures Z1–Z4 identified Z1 and Z2 as two distinct types phylogenetically affiliated with *Symbiodinium* clade A (Fig. 2). Culture Z3 was phylogentically affiliated with *Symbiodinium* clade C, and Z4 contained two *Symbiodinium* types affiliated with *Symbiodinium* clades A and D (Fig. 2).

### Pathogen supernatants have a similar effect on *Symbiodinium* culture Z1

Exposure of the susceptible *Symbiodinium* culture Z1 to supernatants from pathogens P1–P4, resulted in total PS II inactivation (I) in all treatments after 20 minutes (Fig. 1B; p = 0.794). Comparisons of mean I among 16 pathogen-*Symbiodinium* culture combinations (P1–P4 and Z1–Z4) resulted in values ranging between 0 and 1.0 (Table 3).

**Table 3:** PS II inactivation (I) of *Symbiodinium* cultures Z1–Z4 by pathogen supernatants P1–P4.

I; ΔF/F_m′_/10 min^1^	P1^2^	P2^2^	P3^2^	P4^2^
Z1^3^	0.968±0.016	1.0±0	0.962±0.012	1.0±0
Z2^3^	0.587±0.123	0.485±0.048	0.027±0.027	0.707±0.042
Z3^3^	0.659±0.021	0.763±0.013	0.482±0.021	0.675±0.027
Z4^3^	0.034±0.019	0.297±0.036	0.011±0.011	0±0

1 Mean PS II inactivation (I; ΔF/F_m′_±SE; n = 8) was calculated from light adapted effective quantum yields following 10 min of exposure to pathogen supernatants.

2 Pathogen supernatants were obtained by growing pathogen cultures to end logarithmic phase (18 h, 27°C) with shaking (150 rpm).

3 Wells in 96 microtitre plates were inoculated with 1×10^6^ cells ml^−1^ of *Symbiodinium* cultures.

### Pathogen proteolytic activity is inhibited by EDTA and reactivated by ZnCl_2_

EDTA was the most potent inhibitor of proteolytic activity of bacterial supernatants P1–P4 when tested by the asocasein assay [49]–[50] incorporating three standard protease inhibitors (EDTA, 1,10 Pt and PMSF; Fig. S1A). Proteolytic activity was reduced by 98% with the addition of 50 mM EDTA to the supernatant of pathogen P1 (1 h incubation at 30°C; Fig. S1B) and by ∼80%, on average, for pathogens P1–P4 (Fig. S1A). Addition of 10 mM ZnCl_2_ reversed the chelating effect of EDTA and reactivated the proteolytic activity of P1 supernatant to 77% of its original capacity (Fig S1C). This result combined with results from previous screenings [12], which detected the active zinc binding site of a metalloprotease using specific primers targeting the DNA in all pathogens (P1–P4), confirmed the presence of a zinc-metalloprotease. Following the addition of higher ZnCl_2_ concentrations (50 mM and 100 mM), no recovery was detected by the asocasein assay, suggesting that the P1 zinc-metalloprotease requires an optimal concentration of ZnCl_2_ for activity (For more on inhibition of proteolytic activity by excess ZnCl_2_ see Supporting Information Text S1). Other divalent cations (NiCl_2_, MnCl_2_, MgCl_2_, CaCl_2_, CuCl_2_, HgCl_2_ and FeCl_2_) failed to reactivate the P1 zinc-metalloprotease following inhibition by EDTA (data not shown).

### *Symbiodinium* PS II inactivation by pathogen supernatants is inhibited by EDTA

Limited PS II inactivation was observed in all *Symbiodinium* cultures after 45 min when bacterial supernatant P1 was incubated with 50 mM EDTA (Fig. 1C). This was in contrast to strong PS II inactivation when all cultures were exposed to non-chelated supernatants (p<0.01) [mean I (Z1, P1 EDTA, ΔF/F_m′_) = 0.119±0.017, mean I (Z2, P1 EDTA, ΔF/F_m′_) = 0, mean I (Z3, P1 EDTA, ΔF/F_m′_) = 0.267±0.015 and mean I (Z4, P1 EDTA, ΔF/F_m′_) = 0]. The EDTA inhibition of proteolytic activity was not significantly different among the four pathogen supernatants tested (P1–P4; p = 0.566), supporting the hypothesis that they share a common virulence mechanism. Pooling all I (ΔF/F_m′_) data for *Symbiodinium* cultures (Z1–Z4) exposed to four pathogen supernatants (P1–P4) clearly demonstrated that PS II inactivation (I) is caused by bacterial supernatants but was absent in controls in 16 experiments (Fig. 1C; p<0.001). Addition of 50 mM EDTA to pathogen supernatants resulted in significantly lower PS II inactivation (p<0.01). PS II inactivation was not eliminated completely, as shown by levels of I approaching non-EDTA treated supernatants in the first 5 min following exposure (Fig. 1C). However, I in EDTA treatments diminished as time progressed, suggesting that the initial I values were due to EDTA not chelating all zinc cations available in supernatants and therefore preventing complete inhibition of the supernatant activity.

### PS II inactivation is significantly greater when PS II centers are active

Pathogen supernatant-exposure experiments under illumination, equal to the light intensity in the culturing incubator (90 µmol photons m^−2^ s^−1^), resulted in significantly higher I of all *Symbiodinium* cultures (Fig. 1C; p<0.001) in comparison to I calculated from identical control and supernatant exposure experiments that were conducted by measuring quantum yields (F_v_/F_m_) in the dark (Fig. 1D).

### Pathogen supernatants cause *Symbiodinium* PS II inactivation *in hospite*

**Figure 3:**
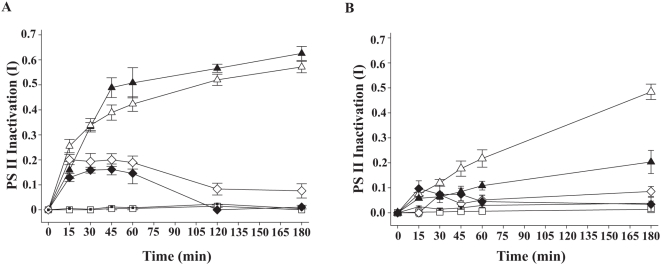
PS II inactivation (I) of *Symbiodinium* in coral juveniles. A. PS II inactivation (I; ΔF/F_m′_) of the coral juvenile *Acropora millepora* hosting *Symbiodinium* clade D by the pathogen supernatants P1 and P3 and four control treatments: P1 supernatant ▴; P3 supernatant ▵; P1 supernatant incubated (1 h 30°C) with 50 mM EDTA ♦; P3 supernatant incubated (1 h 30°C) with 50 mM EDTA ⋄; Dinoflagellate growth medium (F2) □; 1∶1 mix of bacterial growth medium (MB) and dinoflagellate growth medium (F2) ▪. B. PS II inactivation (I; ΔF/F_m′_) of the coral juvenile *Acropora millepora* hosting *Symbiodinium* clade C1 by the pathogen supernatants P1 and P3 and four control treatments: P1 supernatant ▴; P3 supernatant ▵; P1 supernatant incubated (1 h 30°C) with 50 mM EDTA ♦; P3 supernatant incubated (1 h 30°C) with 50 mM EDTA ⋄; Dinoflagellate growth medium (F2) □; 1∶1 mix of bacterial growth medium (MB) and dinoflagellate growth medium (F2) ▪. I; ΔF/F_m′_ was based on measurements of effective light adapted quantum yields. Bars = standard errors. n = 8 measurements per treatment.

As *Symbiodinium* cells may function differently when free-living compared to when *in hospite*, a second bioassay system was developed, comprised of coral juveniles (*Acropora millepora*) harbouring *Symbiodinium* endosymbionts from clades C or D. Juveniles harbouring clade D (JD) *Symbiodinium* and exposed to supernatant from pathogen P1 demonstrated PS II inactivation with mean I (JD, P1, ΔF/F_m′_) = 0.219±0.022 after 10 min and mean I (JD, P1, ΔF/F_m′_) = 0.389±0.030 after 45 min, significantly greater PS II inactivation than found in controls (Fig. 3A; p<0.01). I of JD exposed to P1 continued to increase reaching total inactivation after 7 h. When 50 mM EDTA was added to bacterial supernatants, significantly lower I values were recorded (Fig. 3A; p<0.01). Medium F2 to which 50 mM EDTA were added to test the direct effect of EDTA on coral juveniles had no PS II inactivation effect on juveniles, with mean I (JD, F2+EDTA, ΔF/F_m′_) = 0 after 4 h. *A. millepora* juveniles infected with *Symbiodinium* from clade C1 and exposed to identical treatments demonstrated similar patterns (Fig. 3B).

### Tissue lesions and *Symbiodinium* loss caused by pathogen supernatant

**Figure 4:**
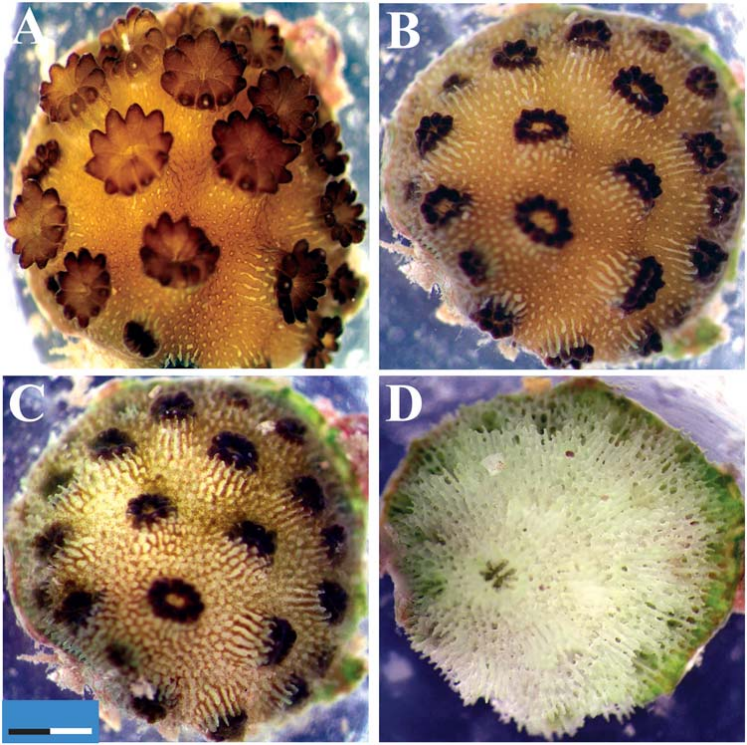
Effect of P1 supernatant on the juvenile coral host, *Acropora millepora*. *A. millepora* juvenile infected with *Symbiodinium* clade D exposed under a dissecting microscope to P1 supernatant. A. Before exposure. B. 2 h following exposure. C. 4 h following exposure. D. 8 h following exposure. Bar = 2 mm (×1.6 enlargement).

*A. millepora* juveniles harboring *Symbiodinium* clade D and exposed to bacterial supernatant (P1 and P3) were observed to pale within minutes following exposure (Fig. 4A). Following addition of bacterial supernatants, juvenile polyps retracted and extended vigorously for a period of 30 sec before becoming irreversibly still. Degradation of the coenosarc tissue (tissue between polyps) was observed (Fig. 4B) and *Symbiodinium* cells were clearly seen separating from juvenile tissue and accumulating beside the host coral. Within 4 h, tissue lesions were observed (Fig. 4C) and by 8 h only skeleton remained (Fig. 4D), corresponding with total PS II inactivation registered by the imaging PAM. *A. millepora* juveniles harboring *Symbiodinium* clade C1 demonstrated similar results when exposed to both P1 and P3 supernatants, while *A. millepora* juveniles treated with supernatants P1 and P3, to which 50 mM EDTA was added, did not show loss of *Symbiodinium* cells or any signs of tissue lesions.

### A biological dose response between P1 Supernatant and Z1 PS II inactivation

**Figure 5:**
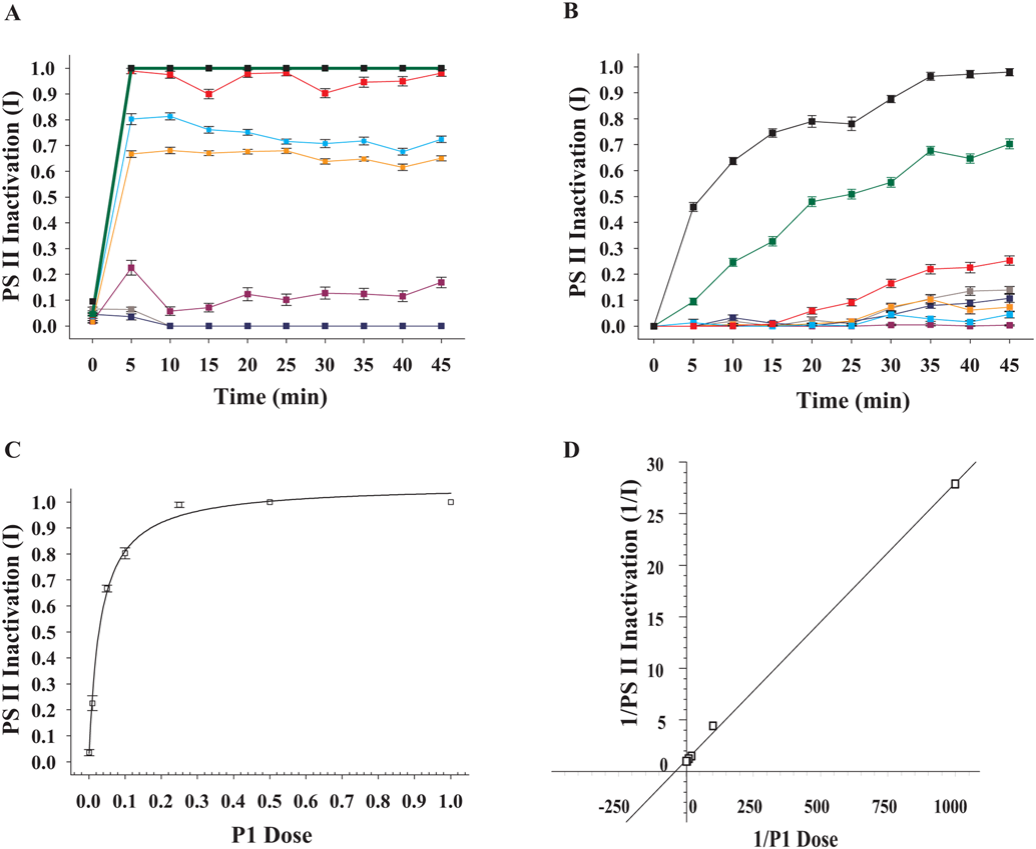
Dose response between P1 supernatant and Z1 PS II inactivation. A. Mean PS II inactivation (I; ΔF/F_m′_) of *Symbiodinium* culture Z1 (2.5×10^5^ cells well^−1^) exposed to dilutions from supernatant P1 stock: 1.0 (black line); 0.50 (green line); 0.25 (red line); 0.1 (azure line); 0.05 (orange line); 0.01 (purple line); 0.001 (blue line), and to control treatment with dinoflagellate growth mediun F2 (grey line). B. Mean PS II inactivation (I; ΔF/F_m′_) of *Symbiodinium* culture Z4 (2.5×10^5^ cells well^−1^) exposed to supernatant P1, to dilutions from P1 supernatant stock: 1.0 (black line); 0.50 (green line); 0.25 (red line); 0.1 (azure line); 0.05 (orange line); 0.01 (purple line); 0.001 (blue line), and to control treatment with dinoflagellate growth medium F2 (grey line). C. Parabolic curved plot for the correlation between P1 supernatant dose (0.001–1.0) vs. mean PS II inactivation (I; ΔF/F_m′_) of *Symbiodinium* culture Z1 following 10 min of exposure to P1. D. Lineweaver-Burk -“like” plot with linear regression for reciprocated P1 supernatant dose vs. reciprocated mean PS II inactivation of Z1 *Symbiodinium* culture following 10 min of exposure. 1/*I max′* is where the linear regression line crosses axis Y, and −1/*km′ is* where regression line crosses axis X. *I max′* = maximum PS II inactivation, and *km′* = the P1 supernatant dilution needed to cause a 50% PS II inactivation (I; ΔF/F_m′_) of *Symbiodinium* culture Z1 following 10 min of exposure. The equation obtained from the linear regression is: Y = 0.027 X+1.071; R^2^ = 0.9991; *km′* = 2.52%. Bars = standard errors. n = 12 measurements per treatment.

Significant PS II inactivation of Z1 was measured by exposure to P1 supernatant concentrations as low as 1% of the original supernatant stock (Fig. 5A; p<0.001), with mean I (Z1, P1, 1%, ΔF/F_m′_) = 0.226±0.028 following 10 min exposure. Total PS II inactivation of Z1 was measured in all P1 concentrations equal and above 25% following a 10 min exposure. In contrast, recovery of photosynthetic activity was detected in Z1 exposed to P1 concentrations of 5% and lower. Full recovery of photosynthetic activity was measured in Z1 cells exposed to 1% and 5% concentrations of P1 following 5 h and 24 h, respectively (p>0.1). In sharp contrast to the susceptible *Symbiodinium* culture Z1, *Symbiodinium* culture Z4 was only affected by higher P1 concentrations, with total PS II inactivation measured for P1 concentrations of 50% and 100% following 2.5 h and 45 min, respectively (Fig. 5B).

Proteolytic activity of bacterial supernatants, measured by the asocasein assay [49]–[50], was found in this study to correlate to culture cell density, with maximum activity measured when cultures reached their end logarithmic growth phase (18 h) and when cell density reached 1×10^9^ cells ml^−1^ (Fig. S2A).

### Enzymatic kinetics supports PS II inactivation by P1 supernatant

To explain the high efficiency of P1 in causing PS II inactivation against in its susceptible Z1 target, we plotted Z1 I (Z1, ΔF/F_m′_) as a factor of P1 dose and obtained a parabolic curve (Fig. 5C) often common in catalytic reactions. Taking the reciprocals of P1 dose and Z1 I resulted in a linear Lineweaver-Burk [51] - ‘like’ plot (Fig. 5D; R^2^ = 0.9991), where apparent km′ represents the effective P1 supernatant concentration causing a 50% PS II inactivation of Z1 following 10 min of exposure. We were unable to determine an actual km and Vmax for P1 due to the fact that the exact concentrations of both the proteolytic peptide and its yet unknown Z1 substrate were not determined. An apparent km′ for Z1 was calculated from the linear regression as km′ = 0.0251, *i.e.*, a P1 supernatant concentration of 2.5% is needed to cause a 50% PS II inactivation of Z1 within 10 min. Taking the reciprocal for P1 dose and Z4 PS II inactivation failed to produce the same linear kinetics.

### Virulent zinc-metalloprotease is a 86.141 kDa *Vibrio* thermolysin

The putative zinc-metalloprotease suspected to cause *Symbiodinium* PS II inactivation and coral tissue lesions was characterised using nano-liquid chromatography peptide separation and mass spectrometry (nano-LC/MS/MS) of proteolytically active bands derived from all pathogen supernatants (P1–P4). These analysis produced signature sequences consistent (by MASCOT and BLAST alignments; see Text S2) with one common bacterial 86.141 kDa pre-propeptide from the family of thermolysin that has been previously identified in other *Vibrio* pathogens, such as *V. cholera* [52] and *V. vulnificus* [53]. Partial protein sequence alignments matched four domains of the common zinc-metalloprotease [54]: the N-terminal domain (PepSY propeptide and YPEB domain), the catalytic domain, the alpha helical domain and the C-terminal domain (Fig. S3).

## Discussion

### Bacterial caused PS II inactivation of *Symbiodinium* photosynthesis

Bacterial causative agents for coral diseases have been identified in previous studies [11]–[12], [55]–[60], including specific virulence mechanisms that enable coral colonization and disease progression [61]–[62], however, this study is the first to investigate the clinical effect of a virulence factor derived from multiple causative agents and applied to multiple targets. Our principal findings demonstrate that PS II inactivation of susceptible *Symbiodinium* cells (Z1) by pathogen supernatants is significantly higher than PS II inactivation of non-susceptible targets (Z2–Z4). Susceptibility of *Symbiodinium* cells to bacterial PS II inactivation was supported by demonstrating a biological dose response [63]. Partial peptide sequencing of proteolytically active fractions derived from WS pathogen supernatants identified a common 86.141 kDa zinc-metalloprotease from the family thermolysin, which is suspected of causing *Symbiodinium* PS II inactivation and coral tissue lesions. Nevertheless, our preliminary exposure trials could not determine the exact process by which bacterial zinc-metalloproteases affect *Symbiodinium* photosynthesis. A similar thermolysin derived from *Bacillus thermoproteolyticus rokko* has been reported to selectively cleave chloroplast outer envelope membrane (OEM) proteins causing PS II photoinhibition [64]. Tests performed with *B. thermoproteolyticus rokko* thermolysin revealed that it can not penetrate through the chloroplast OEM [64], but can affect about 20 OEM polypeptides [65], including components of a protein import apparatus [66] that may indirectly influence PS II performance.

This study identified specific Lineweaver-Burk - ‘like’ kinetics between P1 supernatant and its susceptible Z1 target, suggesting that PS II inactivation of *Symbiodinium* Z1 is potentially the result of a specific bond between an enzyme and its target substrate. Failure to produce similar kinetics between P1 supernatant and a non-susceptible (Z4) target, supports the specificity of this bond and may possibly explain why *Acropora millepore* coral hosts harboring culture Z4 *Symbiodinium* at Nelly Bay (GBR), or *Montipora aequituberculata* colonies harboring Z2 *Symbiodinium* at Davies Reef (GBR) have not been observed with WS disease signs, whereas *M. aequituberculata* colonies from Nelly Bay, which harbor susceptible Z1 *Symbiodinium*, often display WS disease signs. Nevertheless, further studies are needed in order to determine the specific substrate of the zinc-metalloprotease identified in WS coral pathogens in this study. *Symbiodinium* PS II inactivation by low pathogen supernatant concentration (≤5%) was found to be reversible in this study. Recovery of full photosynthetic capacity of *Symbiodinium* cells within 24 h, after short-term PS II inactivation following low concentration exposures, suggests that zinc-metalloprotease damage to PS II might be repairable, as demonstrated for PS II repair by heat shock proteins [67]. Alternatively, PS II inactivation may be caused by enzymatic cleavage of *Symbiodinium* cell membranes, resulting in an irreparable cellular collapse and *Symbiodinium* mortality, as observed by Cervino et al. [68] for yellow blotch/band infections of *Montastraea spp.* corals in the Caribbean. Our study, however, could not find adequate support for this hypothesis, since total PS II inactivation occurred less than 20 sec following exposure to bacterial supernatants. Further studies are needed to examine the pathology of *Symbiodinium* exposed to coral pathogen zinc-metalloproteases. Damage to *Symbiodinium* PS II has been shown to be caused by a variety of factors including light and heat stress [69]–[74] associated with mass coral bleaching [75]–[79], and by numerous bacterial toxins [41], [80] suggesting that PS II damage may result from independent disease aetiologies. In order to test the hypothesis that these factors act in synergism, specific diagnostics must be designed, such as monoclonal antibodies that will register zinc-metalloprotease signals in the field.

In this study, *Symbiodinium* isolates affiliated with clade A were found to be both susceptible and non-susceptible to bacterial PS II inactivation, in contrast to findings by Stat et al. [81], speculating that clade A *Symbiodinium* associations with diseased corals are closer to parasitism than to mutualism than similar associations of corals with clade C *Symbiodinium*. Better knowledge of *Symbiodinium* physiology and disease aetiology will assist in identifying why specific types are more susceptible to bacterial PS II inactivation. Further studies including the cloning and sequencing of the of zinc-metalloprotease genes from WS pathogens will enable validation of our current findings, potentially by utilizing mutant coral pathogen strains that lack the zinc-metalloprotease gene, or by utilizing differential expression and coral pathogen zinc-metalloproteases expressed by a vector system in additional exposure trials [82] aimed at fulfilling Koch's molecular postulates [83].

### Bacterial caused tissue lesions and *Symbiodinium* loss

In this study, visual observations and iPAM measurements of exposed coral juveniles revealed three distinct phases of disease: 1. *Symbiodinium* PS II inactivation; 2. paling of coral tissue through loss of *Symbiodinium* cells; and 3. spread of coral tissue lesions culminating in mortality. These signs, which were expressed by coral juveniles in response to bacterial supernatants, were identical to WS disease signs observed on adult corals in the field [84]–[85] and during pathogen inoculation experiments [12], and further support our previous findings, which identified *Vibrio* pathogens as the primary causative agents of WS's [12]. This is the first study to successfully replicate WS disease signs by using cell free supernatants.

*Vibrio* zinc-metalloproteases are known to perform dual functions similar to the duality of function demonstrated by coral zinc-metalloproteases in this study, *i.e.*, in causing both *Symbiodinium* PS II inactivation and coral tissue lesions. For example, in the human pathogen *Vibrio cholera*, a virulent zinc-metalloprotease has been named hemagglutinin/protease because of its dual capacity to cause both hemagglutination and proteolytic cleavage [53], [86]. The *V. vulnificus* elastase/protease has also been shown to possess dual functions, enhancing vascular permeability and causing hemorrhagic damage [87]–[88]. Numerous studies demonstrate that *Vibrio* zinc-metalloproteases are synthesized as inactive precursors that mature outside the bacterial cell following several processing stages which may alter their function [89], such as the cleavage of a C-terminal 10 kDa peptide from the *V. vulnificus* zinc-metalloprotease (VVP; [90]) by a specific processing protease [91], which mediates effective binding of *V. vulnificus* VVP to its substrate. Future studies will determine whether coral pathogen zinc-metalloproteases undergo a similar maturing process.

### White syndrome is a multifactorial coral disease

Findings from our study support the classification of coral WS as a multifactorial disease with multiple component causes [92]. We found the expression of zinc-metalloprotease by WS coral pathogens to be cell density dependant, with greatest proteolytic activities measured at the end logarithmic phase, when bacterial cell density in cultures reached 1×10^9^ cells ml^−1^. Based on dose response experiments, we calculated that the steady state concentration of coral pathogen derived zinc-metalloprotease required to cause a rapid and irreversible 50% PS II inactivation of susceptible *Symbiodinium* cells following 10 min of exposure is equal to the dose produced by a bacterial concentration of ∼5×10^7^ cells ml^−1^. This calculation, although preliminary, implies that WS disease signs are unlikely to occur in the field unless susceptible populations of *Symbiodinium* cells are exposed to a high concentration of pathogenic bacteria. Thus, the progressing band of exposed coral skeleton typical of WS signs in the field [84]–[85], can be explained by the presence of high densities of pathogens at the interface between exposed skeleton and healthy looking tissue in progressing lesions. In a previous study, we found that *Vibrio* cell densities associated with a lesion interface were more than a 100 times higher than the cell densities found on healthy-looking tissue [12]. In a field study, Roff et al. [93] provided evidence that coral tissues remain photosynthetically active when they are less than 10 cm away from the interface of a progressing WS tissue lesion, suggesting that pathogen zinc-metalloprotease concentrations may be diluted to a non-effective dose away from a progressing tissue lesion interface. Reductions in zinc-metalloprotease concentrations when conditions for optimal pathogen growth are impared may also explain how corals can recover from WS infections.

The findings by Bruno et al. [94], that both temperature and host density influence WS disease prevalence, support the definition of coral WS as a multifactorial disease. These two factors, plus the requirements for primary causative agents [12] at elevated concentrations and for susceptible target *Symbiodinium* types, may all contribute to facilitating WS epizootics. Elevated seawater temperatures have been shown to be a major contributing factor to *Vibrio cholera* pandemics [95] and a necessary factor for triggering the virulence of the coral pathogen *Vibrio shiloi* [96]. Based on our findings, we postulate that optimal temperatures for *Vibrio* WS pathogen growth may contribute directly to the cell density dependant synthesis of zinc-metalloprotease required for infections.

### Are *Vibrio* WS pathogens primary pathogens? Opportunistic pathogens? or secondary pathogens to other unknown causes?

Detection of *Vibrio* strains on both healthy and diseased populations of fish [97], shrimps [98] and corals [24] has led to the conclusion that *Vibrio* infections are opportunistic in nature [99]. The term ‘opportunistic infection’ was first defined by Utz in 1962 relating to fungal infections [100], but has since been modified to include additional pathogen-host interactions, particulary those that can be represented by a ‘damage-response framework’ [101], which defines pathogenicity and host-susceptibility as coupled variant traits. This study identified the variant traits of *Symbiodinium* hosts exposed to a common zinc-metalloprotease. However, it has also been found that the presence of a zinc-metalloprotease gene can be detected in DNA retrieved from non-pathogenic strains, which were unable to cause disease signs in controlled exposure trials [12]. The presence of a zinc-metalloprotease gene in non-pathogenic *Vibrio* strains, suggests that the expression of other virulence genes is necessary for successful infections to occur. Thus we conclude that, although all WS pathogens identified possess a zinc-metalloprotease gene sufficient to cause rapid photoinactivation and coral tissue lesions, not all *Vibrio* strains possessing this gene can be classified as primary causative agents of WS. Genetic studies support the variant traits of *Vibrio* pathogens. From over 200 *V. cholera* serotypes, only a few have been shown to cause cholera pandemics, while others, possessing partial combinations of virulent genes, were shown to cause a gradient of attenuated disease symptoms [102]–[103]. Work by Austin et al. [104] demonstrated that the coral pathogen *V. coralliilyticus* [10]–[12] also affects rainbow trout (*Oncorhynchus mykiss*) and *Artemia nauplii* by causing mortalities in animal models, suggesting it may target multiple species that are not necessarily compromised hosts by possessing broad pathogenicity. In contrast, the virulence of the coral bleaching agent *V. shiloii* [9] was not adequate to infect *Oncorhynchus mykiss* and *Artemia nauplii* [104]. In addition, *V. shiloi* has recently been reported to have stopped infecting its known coral host *Oculina patagonica* in the Mediterranean Sea [105], suggesting a shift in the ‘damage-response framework’ [101], defined by Rosenberg et al. [28] as the ‘hologenome theory of evolution’, *i.e.*, the failure of variant pathogen-host traits to continue producing expected disease signs. Pathogen-coral interactions may be further complicated considering the fact that pathogens in the marine environment perform under different conditions than those confronted by terrestrial agents [106]–[107], and in particular, *Vibrio* coral agents, whose survival strategy might be aimed at specializing in ‘adaptability’ [108], rather than in an obligatory ‘selectivity’ towards specific hosts.

In conclusion, our findings support classifying coral WS's as multifactorial diseases, which are caused by primary *Vibrio* pathogens. Based on findings from this study, *Vibrio* pathogens may be involved in numerous coral disease aetiologies as pathogens of variant traits, and may operate as primary, opportunistic, or as secondary agents. Their ubiquity and modes of action underline the need for further collaborative studies to explore the complexity of roles performed by *Vibrio* zinc-metalloproteases in both coral health and disease.

## Materials and Methods

### Coral pathogens

Four coral pathogen strains (P1–P4; Table 2), previously identified as causative agents for white syndrome diseases (WS's) affecting Indo-Pacific scleractinian corals by fulfilling Henle-Koch's postulates [12], were examined in this study. 16SrRNA gene sequences of all four coral pathogen strains were submitted to GenBank under accession numbers: EU372917, EU372931, EU372934, EU372935 (www.ncbi.nih.nlm.gov/Genbank). All isolates were submitted to the public collection of BCCM/LMG at the Ghent University, Belgium under accession numbers LMG23691, LMG23693, LMG23695, LMG23696, and are available for public acquisition (Table 2).

### Growth curve and proteolytic activities of bacterial supernatants

Each of the four bacterial pathogens (P1–P4) was inoculated into a general heterotrophic bacterial medium, Marine Broth-2216 (Difco, USA) and grown to end logarithmic phase at 27°C with shaking (150 rpm). Tests performed to determine the optimal growth conditions for pathogens P1–P4, demonstrated that culture supernatants expressed the strongest proteolytic activity when incubated for 18 h to end logarithmic phase (Fig. S2A). Bacterial cell density was determined by colony forming unit counts (CFU; described by Sussman et al. [12]) and by constructing a cell density calibration curve of absorbance (595 nn) vs. CFU (Fig. S2B). Absorbance (595 nm) of serial culture dilutions was measured in sterile microtitre 96 well plates (n = 6) using a Wallac Victor 2 1420 multi label counter spectrophotometer (Perkin Elmer, USA). Bacterial supernatants used in exposure experiments were obtained by centrifugation (12,000× g, 20 min, 4°C) and serial filtration through 0.45 µm and 0.22 µm filters (Millipore, USA). These solutions were defined as bacterial supernatants P1–P4, and their protease activity was measured by the asocasein assay [49] as proteolytic units [50], when 1 U = 1000×(OD_450_×CFU^−1^)×10^9^. Protein concentrations in all bacterial supernatants (P1–P4) were determined by the Biorad protein assay (Biorad laboratories, USA). Bacterial supernatant aliquots were stored at −20°C until used.

### Inhibition of proteolytic activity by EDTA and reactivation with ZnCl_2_

Bacterial supernatants (P1–P4) were exposed to treatments with four concentrations of EDTA (5 mM, 10 mm, 25 mM and 50 mM). Triplicate samples of each treatment were incubated for 1 h at 30°C and then tested for proteolytic activity by the asocasein assay [49]–[50]. Control treatments included bacterial supernatant with no EDTA. Treatments of pathogen supernatants inhibited by adding 50 mM EDTA and incubation (1 h, 30°C) were used as negative control treatments in all exposure experiments conducted in this study. The ability to reactivate the proteolytic activity of the P1 pathogen by adding divalent cations was tested by incubating P1 supernatant with 50 mM EDTA (1 h at 30°C ) and adding five concentrations of ZnCl_2_ (5 mM, 10 mM, 25 mm, 50 mM and 100 mM). Samples were incubated for 1 h at 30°C and then tested for proteolytic activity by the asocasein assay [49]–[50] (for more information on the inhibitory effect of excess ZnCl_2_ on the proteolytic activity of pathogen supernatants see Text S1).

### Inhibition by 1,10 Phenanthroline monohydrate (1,10 Pt) and phenyl methylsulfonyl fluoride (PMSF)

1,10 Phenanthroline monohydrate (1, 10 Pt; SIGMA) was dissolved in DDW (Millipore). Pathogen supernatants were incubated for 1 h at 30°C with 1,10 Pt in a final concentration of 5 mM [109]. Proteolytic activity was measured by the asocasein assay [49]–[50]. PMSF (SIGMA), an alkaline serine protease inhibitor, was dissolved in ethanol and incubated for 1 h at 30°C with pathogen supernatants in a final concentration of 5 mM [110]. Following incubation, reactions were assayed for proteolytic activity by the asocasein assay [49]–[50].

### Isolation of *Symbiodinium* cultures from sampled corals

Colonies of *Montipora aequituberculata*, *Acropora tenius* and *Acropora millepora* were collected in sterile containers at Nelly Bay, Magnetic Island, GBR (S19 10′ E 146 52′), an inshore fringing reef. Additional colonies of *Montipora aequituberculata* were collected at Davies Reef, GBR (S18°81′, E147°67′), a midshelf reef located less than 100 km away (Table 1). Coral tissue was removed by airbrush, centrifuged three times (3000× g, 5 min) and resuspended in 0.22 µm filtered SW (25°C). Coral nematocysts were removed by two consecutive filtrations (20 µm; Millipore, USA) using a vacuum pump.

### *Symbiodinium* cultures

F2 dinoflagellate growth medium for *Symbiodinium* was prepared by modification of F2 and Erdschreiber media [111]–[112]. Briefly, seawater supplemented with 4 mg l^−1^ Na_2_HPO_4_, 1 g l^−1^ NaNO_3_, 1 ml l^−1^ from a ×1000 concentrated A_5_+CO micronutrient solution (described by Sussman et al. [113]), 2.5 mg l^−1^ GeO_2_, 80 mg l^−1^ G-Penicillin, 80 mg l^−1^ Streptomycin, 40 mg l^−1^ Amphotericin, 0.4 mg l^−1^ Thiamine-HCl, 2 µg l^−1^ Biotin and 2 µg l^−1^ Vitamin B_12_ (cyanocobalamin). The growth medium was 0.22 µm filtered and stored at 4°C in the dark. Before use, 0.22 µm filtration was repeated.

*Symbiodinium* cultures Z1–Z4 in F2 medium were inoculated into sterile 24 well plates (3 ml per well), covered and sealed. Plates were incubated at 27°C under 12h∶12h light∶dark irradiance (90 pmol photons m^−2^ s^−1^). Cells were inspected daily and contaminated plates were discarded. Prior to experimental exposures, *Symbiodinium* cells were quantified (n = 10) using a Neubauer haemocytometer and adjusted to one concentration (1×10^6^ cells ml^−1^) by adding F2 medium before transferring cultures into sterile 96 well microtitre plates (250 µL per well). An attempt was made to maintain the original *Symbiodinium* populations that were associated with the host coral at the time of isolation rather than to purify and maintain single axenic cultures [114], which would have less ecological relevance when tested for their susceptibility to pathogen supernatants. In order to confirm the taxonomic identity of *Symbiodinium* types in each culture, cloning of *Symbiodinium* DNA was performed at the time of isolation from corals and prior to using the incubated cultures for experimental procedures. Experiments exposing *Symbiodinium* cells to bacterial supernatants and controls were repeated twice to confirm the consistency of results. A full description of treatments is presented in table S1.

### PS II dark adapted quantum yields (F_v_/F_m_) and PS II inactivation (I)

96 well microtitre plates containing *Symbiodinium* cells (1×10^6^ cells ml^−1^) were incubated in the dark (1 h) and centrifuged (5 min at 3000× g). F2 medium was discarded and wells were loaded with treatment solutions. Plates were exposed in a Maxi imaging-pulse-amplitude-modulation (iPAM) fluorometer (Walz, Germany) to a saturation light pulse (Gain = 1–2, Intensity = 1–2, Saturation Pulse = 7) at 5 min intervals and dark adapted PS II quantum yields were calculated by using the formula: Fv/Fm = (Fm−F_0_)/Fm [45], where Fm = maximal fluorescent yield, and F_0_ = Dark fluorescent yield. From F_v_/F_m_ values, PS II inactivation values (I) were calculated as proportions by using the formula: I (F_v_/F_m_ ) = (F_v_/F_m_ at time 0−F_v_/F_m_ at time n)/F_v_/F_m_ at time 0, where 1.0 represented 100% PS II inactivation, following exposure to proteolytically-active and EDTA-inhibited bacterial supernatants and three additional controls (Table S1).

### PS II effective light-adapted yields (ΔF/F_m′_) and PS II inactivation (I)

The identical procedure for sample preparation before measurement of dark adapted yields was repeated before measuring effective light adapted yields. This step confirmed the results obtained from reading photosynthetic inactivation as a proportion of dark adapted yields. Some authors also consider it as a better estimate for photosynthetic function [115], because quantum yields are measured when the cells are photosynthetically active. 96 well microtitre plates were prepared as described above. Each plate was dark adapted first and Fm, F1 and dark adapted quantum yields (Fv/Fm) were recorded at 5 min intervals for a period of 30 min, until consistent levels were obtained. Plates were then centrifuged as described above and returned to the imaging PAM chamber for initial light adapted measurements. An actinic light source of 90 pmol m^−2^ s^−1^ was switched on in the measuring chamber and cultures were exposed to a saturation light pulse at 5 min intervals for a period of 30 min until it was confirmed that readings of effective light adapted quantum yields were stable (Gain = 1–2, Intensity = 1–2, Saturation Pulse = 7). Plates were then removed from the chamber and centrifuged. F2 medium was discarded from the plates and without further delay, plates were returned to the imaging PAM to be loaded with treatment solutions. Plates remained in the imaging PAM chamber under illumination for the entire duration of the experiment. The continuous measurement at 5 min intervals was preferred to the alternative of dark adapting the samples before each single light adapted reading, due to the nature of the experiment. Although photochemical quenching was not fully relaxed, this procedure allowed closer surveillance of the continuous effects of bacterial supernatant on PS II photosynthesis, as it might occur under environmentally relevant conditions, where corals are constantly exposed to light during the day and for longer periods during the summer compared to winter. A similar protocol was used by Schreiber et al. [116] to measure PS II photoinhibition caused by the toxic effects of diuron, suggesting that since quantum yields are calculated from the ratio of fluorescent values before (Ft) and after (Fm′) firing a constant saturation pulse, results are independent of signal amplitudes. According to Schreiber et al. [116], 100 sec intervals between consequent saturation pulses (SP) were sufficient to allow complete reoxidation of Q_A_ and re-establishment of the original Ft levels. Light adapted effective quantum yields (ΔF/F_m′_) were calculated by the formula: (Fm′−Ft)/Fm′ [46], where Fm′ = maximal fluorescent yield under light conditions and Ft = fluorescence before a saturating pulse. PS II inactivation (I) was calculated (as a proportion) from light adapted effective quantum yields (ΔF/F_m′_) as described above. An alternative method for calculating PS II inactivation by comparing PS II quantum yields of treatments with PS II quantum yields of negative controls at corresponding times [116] was tested and provided similar results.

### Taxonomic identities of *Symbiodinium* cultures

DNA was extracted from *Symbiodinium* cultures incubated at 27°C or directly from corals [117] and amplified using primers targeting the ribosomal RNA (rRNA) internal transcribed spacer 1 region (ITS-1; [118]). PCR products were cloned (pCR 2.1 TOPO kit, Invitrogen, CA) and inserts containing plasmid DNA were amplified with a 5′- tet fluorescently labelled ITS-1 forward primer and then screened on a single strand conformation polymorphism (SSCP) gel before sequencing [119]. Retrieved nucleotide sequences (∼360 bp) including the ITS-1 rRNA and its flanking regions were edited using Chromas Lite software version 2.01 (Technelysium) and aligned using ClustalX version 1.83 [120]. Distance matrices were calculated using the DNADIST program in PHYLIP [121] and phylogenetic trees were generated from distance matrices using the neighbour-joining method [122] and Kimura substitution algorithm [123]. Bootstrapping with 1000 replicates was performed using SeqBoot as integrated in PHYLIP [124] and values ≥50% were included for main nodes of the tree. Ribosomal RNA sequences of *Symbiodinium microadriaticum* amplified with the ITS-1 primers and cloned were submitted to GeneBank (www.ncbi.nih.nlm.gov/Genbank) under the accession numbers EU567151–567152, EU567155–567160, EU567167–567168, EU567170, EU567174 (Table 1). Reference *Symbiodinium* types for phylogenetic analyses were obtained from the following authors: AJ311944 [125], AF380532, AF380537, AF380543, AF380546 [118], DQ238587 [126], AY457958 [127], AF334660 [128], AF396629 [129], EF455526, EF455528 [130], out group *Heterocapsa sp.* FK6-D47 AB084097 [131].

### Experimental coral juveniles

Rearing coral juveniles (*Acropora millepora*) and infecting them with *Symbiodinium* clades D and C1 was performed following the protocol of Little et al. [44]; [for details see Supporting Information Text S3]. Individual *A. millepora* juveniles infected with *Symbiodinium* clades D and C1 were placed in 48 well plates and exposed to the following treatments (n = 4): 1. F2 dinoflagellate medium; 2. P1 supernatant diluted 1∶1 with sterile seawater; 3. P3 supernatant diluted 1∶1 with sterile seawater; 4. P1 supernatant diluted 1∶1 with sterile F2 medium, treated with 50 mM EDTA and incubated for 1 h at 30°C; 5. P3 supernatant diluted 1∶1 with sterile F2 medium, treated with 50 mM EDTA and incubated for 1 h at 30°C; 6. bacterial medium (LB) mixed 1∶1 with dinoflagellate medium (F2). All EDTA and non-EDTA treatments were incubated for 1 h at 30°C prior to use. Plates were acclimatized for five days prior to exposure. Measurements and calculation of PS II dark and light adapted quantum yields and PS II inactivation were performed as described above. For measurements of PS II effective light adapted quantum yields (ΔF/F_m′_), an actinic light source of 5 pmol m^−2^ s^−1^, identical to light intensity in the field, was switched on in the measuring chamber of the imaging PAM. Well plates containing *A. millepora* juveniles identical to those exposed to pathogen supernatants and controls under the imaging PAM were exposed and photographed under identical conditions (5-pmol m^−2^ s^−1^, 27°C) at 30 min intervals using a dissecting microscope (×1.6) and a digital camera.

### Pathogen concentration experiment

*Symbiodinium* cultures (Z1 and Z4) were prepared as described above. Pathogen supernatant concentrations were prepared by diluting 0.22 µm filtered P1 supernatant with modified F2 medium to end concentrations of 50%, 25%, 10%, 5%, 1% and 0.1% from original stock. Effective light adapted quantum yield (ΔF/F_m′_) was measured under illumination as described above and PS II inactivation (I) was calculated. I of *Symbiodinium* culture Z1 and concentrations of P1, as proportions of 1.0, were plotted resulting in a parabolic curve (Fig. 5C). Reciprocating data for Z1 PS II inactivation (I) and P1 concentrations resulted in a Lineweaver-Burk [51] - ‘like’ linear plot (Fig. 5D), commonly used to describe the relation between substrate concentration (S) and reaction velocity (V). The term ‘like’ is used in this study, since neither the substrate for bacterial supernatants nor the products of their catalytic activity were determined. It was thus assumed that both supernatant dose and PS II inactivation (I) values are good estimates of S and V. The linear equation (y = ax+b) was used to determine 1/km′, when y = 0 and, with km' defined as the concentration of P1 needed to cause a 50% PS II inactivation (I) of the susceptible *Symbiodinium* culture Z1 within 10 min following exposure.

### Protein sequence retrieval

Bacterial cultures P1–P4 were grown (1.8 L) and crude extracts were derived by ammonium sulphate precipitation [38] and ultra filtration (Amicon 5,000 M MWCO, Millipore, USA) before screening by fast protein liquid chromatography (FPLC). 10 µL from all 72 FPLC-derived fractions were assayed for proteolytic activity by the asocasein assay [49]–[50] and selected samples were run on zymogen gels containing 0.1% Na-casein as substrate [132]. Active fractions were re-run on a 12% SDS-PAGE [133] and bands were excised for nano-liquid chromatography peptide separation and mass spectrometry. LC/MS/MS data were searched using Mascot (Matrix Science, London, UK) and bacterial entries in the NCBI non-redundant protein database [134]. Additional information on FPLC and nano-LC/MC/MC protocols appears in Supporting Information Text S2.

### Statistical Analysis

Means and standard errors (SE) for bacterial colony forming unit (CFU) counts, for absorption readings (bacterial cell density and proteolytic activity), for PS II dark adapted quantum yields and light adapted effective quantum yields were compared among treatments using One-Way ANOVA (Statistica, StatSoft, Inc. USA). CFU counts are presented in this study using logarithmic scales. Means and standard errors (SE) for PS II inactivation (as a proportion of 1.0) in all exposure experiments (treatments and controls) were compared using multivariate repeated measures MANOVA (Statistica, StatSoft, Inc. USA), which does not rest on the assumption of sphericity and compound symmetry [135]. Four multivariate tests of significance were applied (*Wilks' Lambda*, *Pillai-Bartlett Trace*, *Hotelling-Lawley Trace*, and *Roy's Largest Root*) with non-significant results used to overrule any previous assumptions of statistical significance. Significant results were determined when α≤0.05.

## Supporting Information

Figure S1 Inhibition of proteolytic activity of *Vibrio* pathogens. Legend for Fig. S1 can be found in Supporting Information file S1
(0.49 MB EPS)

Figure S2 Pathogen P1 growth conditions. A. Mean bacterial cell density (absorbance 595 nm) vs. incubation time (27°C with shaking at 150 rpm) appears in grey, and mean proteolytic activity in Units determined by the asocasein assay (black line) vs. incubation time. B. Calibration curve for cultures of pathogen P1: mean cell density (CFU) vs. mean cell density (absorbance 595 nm). n = 6 measurements per treatment. Additional information on Fig. S2 can be found in Supporting Information file S2.
(1.80 MB EPS)

Figure S3 Zinc-metalloprotease conserved domains. Domains of a 86.141 kDa pre-propeptide, a zinc-metalloprotease derived from coral pathogen supernatants (P1–P4). Propeptide (pink); PepSY (propeptide and YPEB domain; yellow); Catalitic domain (green); α-helical domain (azure); C-terminal domain (red). P1–P4 partial protein sequence alignments (BLAST/MASCOT) matched sequences of four conserved domains from previously identified *Vibrio* zinc-metalloproteases: Pathogen supernatant P3 - PepSY (propeptide and YPEB domain; yellow); Pathogen supernatant P3 - Catalitic domain (green); Pathogen supernatant P2–P4 - α-helical domain (blue); Pathogen supernatant P1 - C-terminal domain (red). Additional information on Fig. S3 can be found in Supporting Information file S3.
(1.01 MB EPS)

Text S1 Effect of ZnCl2 on proteolytic activity
(0.03 MB DOC)

Text S2 Protein sequence retrieval
(0.03 MB DOC)

Text S3 Rearing coral juveniles
(0.03 MB DOC)

Table S1 Bioassay of *Symbiodinium* cultures; treatment allocation. ^1^ Each 96 well micro titre plate was loaded with equal aliquots from three Symbiodinium cultures (250 µL = 1×106 cells ml^−1^). Treatments (250 µL per well) were added at experimental begin. Plates were rotated by 180° during the experiment in order to verify that PS II yield readings from the edges of the microtitre plates were identical to those obtained from its inner parts. ^2^ Treatments with 50 mM EDTA were incubated for 1 h at 30°C before being used for exposure experiments. Treatments without EDTA were incubated under the same conditions (1 h, 30°C).
(0.03 MB DOC)

Supporting Information File S1 Supporting Information file S1 contains the legend of Figure S1
(0.03 MB DOC)

Supporting Information File S2 Pathogen P1 growth conditions
(0.02 MB DOC)

Supporting Information File S3 Zinc-metalloprotease conserved domains
(0.02 MB DOC)
