# Supplementary material for: Vibrio Zinc-Metalloprotease Causes Photoinactivation of Coral Endosymbionts and Coral Tissue Lesions
Source: PLoS One. 2009 Feb 19;4(2):e4511. doi: 10.1371/journal.pone.0004511 (PMC2637982; doi:10.1371/journal.pone.0004511)
Supplement: Text S1 — Effect of ZnCl2 on proteolytic activity (0.03 MB DOC) [file pone.0004511.s004.doc]

**Supporting Information Text S1: Effect of ZnCl_2_ on proteolytic activity**

Inhibition of pathogen P1 supernatant by EDTA reached 98% following exposure to 50mM EDTA (Fig. S1B). At lower concentrations, inhibition dropped to 87%, when P1 supernatant was exposed to a 5mM concentration and 91% and 96% when exposed to 10mM and 25mM, respectively. When 10mM ZnCl_2_ were added to EDTA-inhibited samples, P1 supernatant recovered 77% of its proteolytic activity prior to EDTA inhibition (Fig. S1C). When treated with other ZnCl_2_ concentrations, recovery was significantly lower, with 61% recovery following the addition of 5mM ZnCl_2_ and 35% recovery following the addition of 25mM ZnCl_2_. Following the addition of higher ZnCl_2_ concentrations (50mM and 100mM), no recovery was detected by the asocasein assay [49-50], suggesting that the P1 harbors a zinc-metalloprotease that needs an optimal concentration of ZnCl_2_ for activity (Fig. S1C). To test this, P1 supernatant was incubated with five concentration of ZnCl_2_ (5mM, 10mM, 25mm, 50mM and 100mM) without prior inhibitory treatment with EDTA. Results demonstrated that the proteolytic activity of P1 supernatant was significantly affected by excess ZnCl_2_ in all treatments, with a 95% loss of activity measured in the 10mM-100mM concentrations and 81% activity-loss when 5mM ZnCl_2_ were added to the supernatant (data not shown). Similar inhibition was demonstrated when exposing the *H. pylori* zinc-metalloprotease to zinc concentrations above 1mM [49]. Other zinc peptides are also inhibited by excess zinc (competitive inhibition), which is proposed to result from the formation of zinc monohydroxide multidentate complexes which bridge the catalytic zinc to a side chain on the active site [136].
