# Supplementary material for: Vibrio Zinc-Metalloprotease Causes Photoinactivation of Coral Endosymbionts and Coral Tissue Lesions
Source: PLoS One. 2009 Feb 19;4(2):e4511. doi: 10.1371/journal.pone.0004511 (PMC2637982; doi:10.1371/journal.pone.0004511)
Supplement: Text S2 — Protein sequence retrieval (0.03 MB DOC) [file pone.0004511.s005.doc]

**Supporting Information Text S2:** **Rearing coral juveniles**

Gametes were collected following spawning of eight (hermaphroditic) colonies of *Acropora millepora*, during the spawning event in October, and mixed together for fertilization.  Fertilized larvae were raised in filtered seawater in two separate 500 L tanks in a temperature controlled (27° C) aquaria room. When the larvae were elongated and searching for settlement sites (four days after spawning), preconditioned and autoclaved terra-cotta tiles (preconditioned for six weeks at Magnetic Island, GBR) were placed on the bottom of the two 500 L tanks to provide settlement surfaces.  *Symbiodinium* clade strains D and C1 were obtained from adult *A. millepora* and *A. tenuis* colonies from Magnetic Island, respectively, by airbrushing the coral tissue and isolating *Symbiodinium* cells out of the coral-*Symbiodinium* slurry by centrifugation (5min. 3500 x g). These *Symbiodinium* cells were offered at 10^8^ cells tank ^-1^ three and five days after spawning and were acquired by newly settled juveniles. The terra-cotta tiles were attached to a fringing reef (Nelly Bay, Magnetic Island, GBR) in a zone where *Acropora spp.* were present and were then collected nine months after settlement (in July) for experimental exposure. Tile racks were removed from the reef and carried in large containers to the outdoor aquaria at the Australian Institute of Marine Science (AIMS). Individual tiles were separated and placed to recover in outdoor tanks with a flow-through seawater system. Following five days, individual juveniles were scraped from the tiles and placed in sterile 48 well plates. These plates were further suspended in flow-through tanks for recovery until juveniles attached to the bottom surface of the wells. In this setup, juveniles experimentally infected with either *Symbiodinium* D or C1 cultures originated from crosses involving the same parent corals, thereby minimizing potential host genetic differences that may influence the physiology of the holobiont (host-symbiont partnership).
