# Supplementary material for: Vibrio Zinc-Metalloprotease Causes Photoinactivation of Coral Endosymbionts and Coral Tissue Lesions
Source: PLoS One. 2009 Feb 19;4(2):e4511. doi: 10.1371/journal.pone.0004511 (PMC2637982; doi:10.1371/journal.pone.0004511)
Supplement: Text S3 — Rearing coral juveniles (0.03 MB DOC) [file pone.0004511.s006.doc]

**Supporting Information Text S3: Protein sequence retrieval**

Proteins in a total volume of 100 ml of supernatant derived from each of four pathogen cultures were precipitated by slowly adding ammonium sulphate to achieve the final % saturations of 20%, 40%, 60%, 80% and 100%. This procedure was undertaken to determine the optimal ammonium sulphate concentration needed to precipitate bacterial proteolytic enzymes from their corresponding supernatants without compromising proteolytic activity. Samples were stirred for 2h and then centrifuged at 5000 x g for 1h at 4ºC. Supernatants were discarded and the remaining precipitate was diluted by adding 200µl of 0.22µm-filtered phosphate buffered saline (PBS: 10mM Potassium Phosphate 150mM NaCl pH7.4). Samples were tested for proteolytic activity by the asocasein assay [49-50] and the optimum % saturation of ammonium sulphate was chosen for the production of higher concentrations of active crude extracts from all pathogen supernatants. Pathogens P1-P4 measured the highest proteolytic activity by the asocasein assay when precipitated in a 20% saturation of ammonium sulphate.

For the production of proteolytic active crude extracts, 1.8L volumes of bacterial cultures were incubated and crude extracts, diluted in 45ml 0.22µm-filtered PBS (7.4 pH), were derived (as described above). Each sample was then ultra-filtered using three Amicon 5,000M MWCO ultra centrifugal filter devices (Millipore, USA). Samples were centrifuged at 4000 x g in a swinging bucket Alegra X-15R centrifuge (Beckman Coulter, CA) at 4ºC for 45 min to derive a total final crude extract volume of 600µL from each pathogen supernatant, corresponding to a x3000 concentration of the original pathogen cultures. 1µL of each sample was then diluted in 99µL of 0.22µm-filtered DDW (Millipore, USA) and tested to confirm proteolytic activity by the asocasein assay [49-50]. Protein concentrations were measured with a protein assay kit from Bio-Rad and samples were frozen at -80ºC and sent to the Australian Proteome Analysis Facility (Macquarie University NSW, Australia) for fast protein liquid chromatography (FPLC) analysis. The samples were run on a Superdex 75 10/300 GL Column and the buffer used was PBS (pH7.4). The column was tested with low molecular weight standards (GE Biosciences) before the sample was injected (Albumin 67kDa, Ovalbumin 43kDa, Chymotrypsinogen A 25kDa and Ribonuclease A 13.7 kDa). The samples were prepared by filtering through a 0.45µm cellulose acetate microcentrifuge filter system unit at 10,000 x g for 1 min. The method flow rate was 0.5ml for 70 min and fraction size was 400uL (48 seconds). The sample was run on a Waters Alliance HT 2795 system with a PDA detector and Gilson FC204 fraction collector. 10µL from all 72 fractions from each sample were assayed for proteolytic activity by the asocasein assay [49-50] and results were superimposed on the 280nm chromatograms generated for each sample for fraction selection. Selected samples were run on zymogen gels containing 0.1% Na-casein co-polymerized in gels as substrate revealing zones of hydrolysis. 15 active fractions from all four pathogens were re-run on a 12% SDS-PAGE following the method by Laemmli [133]. 15 Bands were excised using a sterile blade and sent to the Australian Proteome Analysis Facility for nano-liquid chromatography peptide separation and mass spectrometry. Gel plugs were de-stained followed by tryptic digestion for 16h at 37ºC. Digested peptides were separated by nano-LC using a CapLC system (Agilent 1100 Series, Agilent Technologies, Germany). Sample (39μL) was injected onto a peptide trap (Michrome peptide Captrap) for pre-concentration and desalted with 0.1% formic acid at 10μL/min. The peptide trap was then switched into line with the analytical column containing C18 RP silica (SGE ProteCol C18, 300A, 3μm, 150μm x 10 cm). Peptides were eluted from the column using a linear solvent gradient, with steps, from H_2_O:CH_3_CN (95:5; + 0.1% formic acid) to H_2_O:CH_3_CN (20:80, + 0.1% formic acid) at 500nLmin^-1^ over a 45 min period. The LC eluent was subject to positive ion nano flow electrospray analysis on an Applied Biosystems QSTAR XL mass spectrometer (ABI, CA, USA). The QSTAR was operated in an information dependant acquisition mode (IDA). In IDA mode a TOFMS survey scan was acquired (m/z 400-2000, 1.0s), with the four largest multiple charged ions (counts >25) in the survey scan sequentially subjected to MS/MS analysis. MS/MS spectra were accumulated for 1 s (m/z 50-2000).
