# Supplementary material for: Vibrio Zinc-Metalloprotease Causes Photoinactivation of Coral Endosymbionts and Coral Tissue Lesions
Source: PLoS One. 2009 Feb 19;4(2):e4511. doi: 10.1371/journal.pone.0004511 (PMC2637982; doi:10.1371/journal.pone.0004511)
Supplement: Table S1 — Bioassay of Symbiodinium cultures; treatment allocation. 1 Each 96 well micro titre plate was loaded with equal aliquots from three Symbiodinium cultures (250 µL = 1×10^6^ cells ml−1). Treatments (250 µL per well) were added at experimental begin. Plates were rotated by 180° during the experiment in order to verify that PS II yield readings from the edges of the microtitre plates were identical to those obtained from its inner parts. 2 Treatments with 50 mM EDTA were incubated for 1 h at 30°C before being used for exposure experiments. Treatments without EDTA were incubated under the same conditions (1 h, 30°C). (0.03 MB DOC) [file pone.0004511.s007.doc]

**Supporting Information Table S1: Bioassay of *Symbiodinium* cultures; treatment allocation**

|  | **1** | **2** | **3** | **4** | **5** | **6** | **7** | **8** | **9** | **10** | **11** | **12** |
| --- | --- | --- | --- | --- | --- | --- | --- | --- | --- | --- | --- | --- |
|  | ***Symbiodinium* culture^1^** | | | | ***Symbiodinium* culture^1^** | | | | ***Symbiodinium* culture^1^** | | | |
| **A** | **F2 dinoflagellate growth medium** | | | | | | | | | | | |
| **B** | **F2 growth medium + 50mM EDTA^2^** | | | | | | | | | | | |
| **C** | **Pathogen supernatant** | | | | | | | | | | | |
| **D** | **F2 dinoflagellate growth medium (same as A)** | | | | | | | | | | | |
| **E** | **Pathogen supernatant + 50mM EDTA^2^** | | | | | | | | | | | |
| **F** | **Pathogen supernatant (same as C)** | | | | | | | | | | | |
| **G** | **1:1 of F2 dinoflagellate growth medium + MB bacterial growth medium** | | | | | | | | | | | |
| **H** | **Pathogen supernatant + 50mM EDTA^2^ (same as E)** | | | | | | | | | | | |
