# Supplementary material for: Vibrio Zinc-Metalloprotease Causes Photoinactivation of Coral Endosymbionts and Coral Tissue Lesions
Source: PLoS One. 2009 Feb 19;4(2):e4511. doi: 10.1371/journal.pone.0004511 (PMC2637982; doi:10.1371/journal.pone.0004511)
Supplement: Supporting Information File S1 — contains the legend of Figure S1 (0.03 MB DOC) [file pone.0004511.s008.doc]

**Figure S1. Inhibition of proteolytic activity of *Vibrio* pathogens**

**A.** Mean proteolytic activity of pathogens P1-P4 supernatants determined by the asocasein assay and inhibited by 50mM EDTA, 5mM 1, 10 Pt and 5mM PMSF. Pathogen supernatant P1 ■; pathogen supernatant P2 ■; pathogen supernatant P3 ■; pathogen supernatant P4 ■; negative control □. **B.** Mean proteolytic activity of pathogen P1 supernatant (Units) determined by the asocasein assay ■, and inhibited by incubation of P1 supernatants (1 h, 30ºC) in concentrations of EDTA: 5mM EDTA ■; 10mM EDTA ■; 25mM EDTA ■; 50mM EDTA; negative control □. **C.** Effect of ZnCl_2_ on restoring the proteolytic activity of P1 supernatants inhibited by incubation (1 h, 30ºC) with 50mM EDTA. Mean proteolytic activity of P1 supernatant (Units) ■; Mean proteolytic activity of P1 supernatant incubated with 50mM EDTA (1 h, 30ºC) ■ and 100mM ZnCl_2_ ■; 50mM ZnCl_2_ ■; 25mM ZnCl_2_ ■; 10mM ZnCl_2_ ■; 5mM ZnCl_2_ □. Negative control treatments were prepared by boiling *E. coli* supernatant (15 min, 100ºC). Bars = standard errors. n = 6 measurements per treatment.
