# Supplementary material for: Vibrio Zinc-Metalloprotease Causes Photoinactivation of Coral Endosymbionts and Coral Tissue Lesions
Source: PLoS One. 2009 Feb 19;4(2):e4511. doi: 10.1371/journal.pone.0004511 (PMC2637982; doi:10.1371/journal.pone.0004511)
Supplement: Supporting Information File S2 — Pathogen P1 growth conditions (0.02 MB DOC) [file pone.0004511.s009.doc]

**Supporting Information for Fig. S2 – Pathogen P1 growth conditions**

P1 bacterial cultures were grown as described in M&M. For the construction of growth curves for pathogen cultures including the corresponding pathogen-supernatant proteolytic activity (Fig. S2A), samples were taken from cultures each hour during growth for measuring bacterial cell density and each 2 h for determining proteolytic activity (Units) of supernatants by the asocasein assay [49-50]. In order to construct a calibration curve (Fig. S2B), cultures were sampled (1 ml) and streaked in triplicates on agar plates containing Marine Agar (Difco, USA; as described by Sussman et al. [12]). Bacterial cell densities (absorbance 595 nm) of each sample were calculated as described in M&M.
