# Supplementary material for: Vibrio Zinc-Metalloprotease Causes Photoinactivation of Coral Endosymbionts and Coral Tissue Lesions
Source: PLoS One. 2009 Feb 19;4(2):e4511. doi: 10.1371/journal.pone.0004511 (PMC2637982; doi:10.1371/journal.pone.0004511)
Supplement: Supporting Information File S3 — Zinc-metalloprotease conserved domains (0.02 MB DOC) [file pone.0004511.s010.doc]

**Supporting Information for Fig. S3 - Zinc-metalloprotease conserved domains**

Partial zinc-metalloprotease sequences were obtained using nano-liquid chromatography peptide separation and mass spectrometry (nano- LC/MS/MS). Data were searched using Mascot and bacterial entries in the NCBI non-redundant protein database [134]. Four out of five domains of a common 86.141 kDa *Vibrio* pre-propeptide were identified and close matches are presented in boxes.
